# Effect of Selenium on the Growth and Lipid Accumulation of *Yarrowia lipolytica* Yeast

**DOI:** 10.1007/s12011-020-02266-w

**Published:** 2020-07-06

**Authors:** Marek Kieliszek, Marianna Dourou

**Affiliations:** 1 Department of Food Biotechnology and Microbiology, Institute of Food Sciences, Warsaw University of Life Sciences—SGGW, Nowoursynowska 159 C, 02-776 Warsaw, Poland; 2 Unit of Microbiology, Division of Genetics, Cell and Developmental Biology, Department of Biology, University of Patras, 26504 Patras, Greece

**Keywords:** Selenium, Y*arrowia lipolytica*, Detoxification, Lipid accumulation, Fatty acid composition, Yeast

## Abstract

Nowadays, there is an increase attention on the effect of selenium (Se) on metabolic processes of microorganisms. Strains belonging to the genus of *Yarrowia* are of great biotechnological interest for various industries. In this study, we evaluated the effect of 10 mg/L of Se on the growth and lipid production of two *Yarrowia lipolytica* strains: the ACA DC 50109 and one more with increased oleagenicity, derived after ALE methodology (referred here as *Y. lipolytica* ALE_70). The presence of Se in the growth medium negatively affected both cell mass production and total lipid accumulation, for both *Y. lipolytica* strains. Fractionation of total lipids showed an inhibition on neutral lipid (NL) synthesis and consequently, an increase of polar lipids (glycolipids plus sphingolipids, and phospholipids) on the lipids of the Se-enriched ACA DC 50109 strain; however, the NL/polar ratio of the Se-enriched ALE_70 indicated that Se, apart from the inhibition of NL synthesis, provoked also the accumulation of polar lipids in this strain. In addition, the fatty acid (FA) composition was differently affected by Se. Se-enriched total lipids of the ALE_70 strain were enriched in linoleic acid (C18:2 n-6), which resulted in increase of the unsaturated index. On the other hand, Se-enriched lipids of the ACA DC 50109 strain were more saturated, as the percentage of palmitic (C16:0) and stearic (C18:0) acids increased in the total FAs. Moreover, it seems that Se influenced the activity or the expression of desaturases and elongase in both strains. Finally, the supplementation of growth medium with Se affected cell morphology, as well as the size and distribution of lipid droplets inside the yeast cells. According to our opinion, Se caused stress conditions and the consequence of that was the occurrence of metabolic disorders that affected cell mass, lipid content, and/or morphological structures. The results of the present study suggest that further research should be carried out to understand the background of the lipogenesis process in yeast cells cultured under stress conditions.

## Introduction

Selenium (Se) is a metalloid element essential for humans, and animals, having antioxidant and anticancer properties, while contributing to the protection and the proper function of the heart, the immune system, and the thyroid glands [1–3]. Se acts as a cofactor of many enzymes (e.g., glutathione peroxidase, deiodinase iodothyronine, and thioredoxin reductase) and plays a significant role in metabolic processes related to selenoprotein formation and function, in redox signaling processes, oxidation-reduction reactions, and in the general protection of an organism against oxidative stress—a state in which the oxidizing potential increases to a level that threatens the stability of the cellular structures [4]. However, Se bioavailability and its effect (i.e., beneficial or toxic) strongly depend on both its chemical form and dose [5]. Se is absorbed by humans through diet, as it is a common element in nature, found in the atmosphere, water and soil, and thus in a variety of foods [6]. In some regions of the world, it has been reported that Se concentration in soil, and consequently in foods, is insufficient to support dietary needs. Therefore, nowadays the supplementation of human diet with Se-enriched microbial cell mass (mostly by the yeast *Saccharomyces cerevisiae*) or with beverages produced by Se-enriched yeasts is suggested as an alternative source for the necessary daily dose of Se [1, 7, 8]. On the other hand, Se is present on contaminated waters and soil. Thus, microorganisms (mostly bacteria), which are able to metabolize certain Se-containing compounds for their cellular processes, could potentially lead to detoxification processes of these wastes [9]. However, Se may have toxic effects also on microorganisms, depending yet again on the dose and its form, as well as on the tolerance/sensitivity of the individual strain.

Se-enriched yeast is achieved by cultivating the yeast in a growth medium containing inorganic Se (selenite or selenate). During optimal growth conditions [10], Se can be transferred within the cell wall-membrane system by biopolymers (e.g., proteins, phospholipids, or polysaccharides) present on the yeast cell or it can be intracellularly accumulated through active transport [11]. Finally, the inorganic form of Se is converted to organic, mostly into selenomethionine [12, 13]. However, when high doses of Se are used, oxidative stress is induced. This phenomenon results in the development of metabolic disorders (e.g., increase in reactive oxygen species (ROS) formation, Se stress-induced peroxisome proliferation, inactivation of antioxidant enzymes), which in turn intensifies the processes affecting the degradation of intracellular organelles and the disorganization of the cell membrane. All these ultimately cause dramatic effect on cell viability, cell cycle, protein synthesis, and DNA integrity [14–16]. Worthy of mentioning is that the influence of Se in the microbial central metabolism has not yet been fully understood, while the effect of Se on the biochemical and physiological processes of oleaginous microorganisms is not yet explored. To the best of our knowledge, Čertík et al. [17] reported that a remodeling process of membrane lipid composition in red yeasts occurred, as a response to Se presence.

Oleaginous microorganisms (i.e., yeasts, fungi, bacteria, and microalgae) are able to accumulate oil (triacylglycerols (TAGs)), so-called single cell oil (SCO) to more than 20% w/w in their dry cell mass [18]. SCOs having an exceptional structure (e.g., similar to that of exotic fats, such as cocoa and shea butter) [19, 20] or fatty acid (FA) composition (i.e., containing polyunsaturated fatty acids (PUFAs)) [21] are of great biotechnological interest for food, pharmaceutical, and chemical industries [22, 23]. Besides, SCOs produced mostly by yeasts, present in general similar FA composition with that of common plant oils; therefore, they may be considered feedstock in the biodiesel manufacture [24, 25]. However, the production cost of microbial oil is still too high, due to the limited productivity of oleaginous microorganisms [26]. Therefore, a variety of agro-industrial and aquaculture by-products of low acquisition cost, as well as lignocellulosic material, are proposed as substrates for oleaginous microorganisms [25, 27–30]. In addition, new strategies using either microorganisms genetically modified or derived after adaptive laboratory evolution approaches, capable of producing cell mass and SCOs in increased quantities, are recently suggested [23, 31, 32]. An example of waste products that are used is potato juice. Such a product contains mineral compounds, protein, vitamins, and glycoalkaloids [33].

Oleaginous yeasts belong to the genera of *Yarrowia*, *Candida*, *Rhodotorula*, *Cryptococcus*, *Apiotrichum*, and *Lipomyces* [34]. Among them, the genus of *Yarrowia* has earned increasing attention by researchers, especially the non-conventional strain of *Yarrowia lipolytica*. *Yarrowia lipolytica*, possessing a rich enzymatic arsenal, has the ability to synthesize a variety of valuable cellular metabolites (e.g., SCOs, enzymes, proteins or recombinant proteins, polyols) and abundant lipid-free cell material and to excrete organic acids, while cultivated on hydrophobic (e.g., *n*-alkanes, oils, fats, and FAs) or hydrophilic substrates (e.g., glucose, raw glycerol, and other industrial and agro-industrial wastes) [35–38]. Thanks to its well-studied biochemistry of lipid accumulation and degradation, genetic profile, and the tools for its genetic manipulation, *Y. lipolytica* is characterized as a model microorganism for oleaginous microorganisms [23].

Three distinct physiological phases characterize the life cycle of oleaginous heterotrophic microorganisms, when cultivated in media with glucose as carbon and energy source and high C:N ratio: (a) the balanced growth phase, (b) the oleaginous phase, and (c) the phase of reserve lipid degradation [23, 39]. During the balanced growth phase, microorganisms accumulate mainly polar lipids to support the construction of cell membranes [39]. In addition, the biosynthesis of precursors and nucleotides involved in the production of energy (i.e., NADH from the Embden-Meyerhof-Parnas (EMP) pathway) or in the production of reducing power (i.e., NADPH from the pentose phosphate pathway (PPP)) occurred [23]. Lipogenesis (the de novo accumulation of TAGs) occurred after the exhaustion of one or more essential nutrient from the growth environment during the oleaginous phase [19], and storage lipids are deposited in the form of lipid droplets (LDs) in the cytosol. Nitrogen exhaustion from the growth medium leads eventually to the disturbance of the tricarboxylic acid cycle by the inhibition of the mitochondrial isocitrate dehydrogenase, a required condition for the onset of lipogenesis. Citrate (characterized as precursor for lipid synthesis) from the mitochondrion, after reaching a critical value, is excreted to the cytoplasm and finally it is cleaved to oxaloacetate and acetyl-CoA. Acetyl-CoA is converted into malonyl-CoA, which is further converted into long-chain acyl-CoA [23]. Although, usually reducing power in the form of NADPH, required for FA synthesis, is provided either/both from PPP or/and from malic enzyme reaction, in *Y. lipolytica*, the main donor of NADPH is PPP [40], while recently, mannitol cycle is also suggested [38]. Then, the long-chain acyl-CoA is transported to the endoplasmic reticulum and esterified with glycerol-3P (G3P), generating structural and storage lipids. G3P is generated by the activity of triosephosphate isomerase (TSI), which catalyzes the reversible interconversion of G3P and DHAP. Finally, depending on the environmental conditions, and after the exhaustion of the carbon source in the growth medium, storage lipids may be degraded, being used as energy source for maintenance purpose or acting as intracellular carbon source supporting cell proliferation [39]. The released FAs from lipid degradation process are catabolized via β-oxidation process towards acetyl-CoA, in the peroxisomes. During FA degradation, lipid remodeling has also been reported [39].

The aim of this current investigation is to study the effect of Se, after its inclusion as a component in the growth medium, on lipogenesis (i.e., accumulation of TAGs and distribution of lipid fractions), FA profile of total lipids, and oxidative stress on *Y. lipolytica*, so as to understand its role on the lipid metabolism and detoxification processes. Our hypothesis is based on the fact that FA biosynthesis requires high quantities of reducing power. Consequently, organisms that accumulate high lipid percentages are highly exposed to oxidative stress, as reducing power is mostly driven to FA biosynthesis, while a smaller amount remains available to be used for protection against oxidative stress. Therefore, two strains of *Y. lipolytica*, which differ each other to the lipid accumulating capacity, were cultivated in media with and without the incorporation of Se.

## Materials and Methods

### Yeast Strains

Two strains of *Yarrowia lipolytica* were used in the current investigation: the ACA DC 50109 (culture collection of Agricultural University of Athens) and one more with increased oleagenicity, derived after the propagation of the ACA DC 50109 strain for 70 generations when adaptive laboratory evolution methodology as described by Daskalaki et al. [32] was applied and referred here as “ALE_70” (culture collection of University of Patras). The stains were stored at 4 ± 1 °C on potato dextrose agar (Himedia, Mumbai, India) and regularly sub-cultured.

### Culture Conditions

Deionized water filtered using a Milli Q system (Millipore, France) was used for the media and Se solution preparation. Cultures were performed in 250-mL Erlenmeyer flasks containing 50 mL of a medium having the following composition (in g/L): glucose, 70.0 (AppliChem, Darmstadt, Germany); Na_2_HPO_4_, 12.0 (AppliChem); KH_2_PO_4_, 12.0 (AppliChem); yeast extract, 3.0 (Conda, Madrid, Spain); FeCl_3._6H_2_O, 0.1 (AnalaR NORMAPUR®, Poole, England); CaCl_2_.2H_2_O, 0.1 (Carlo Erba, Rodano, Italy); ZnSO_4_.7H_2_O, 0.001 (Merck, Darmstadt, Germany); CuSO_4_.5H_2_O, 0.0001 (AnalaR NORMAPUR®); Co(NO_3_)_3._3H_2_O, 0.0001 (Merck); and MnSO_4_.5H_2_O, 0.0001 (Fluka, Steinheim, Germany). In some cases, the media were supplemented with sterile Na_2_SeO_3_ solution (1000 mg Se^4+^/L; Sigma-Aldrich, Warsaw, Poland) in order to reach a final Se concentration of 10 mg/L. The media and aqueous Na_2_SeO_3_ salt solutions were sterilized at 121 °C for 20 min. The medium pH after sterilization was 6.0 ± 0.3 and remained almost unchanged during cultivations, due to its high buffer capacity [39].

After sterilization, the cultures were inoculated with 1 mL of a 48-h pre-culture (3.0–4.0 × 10^5^ cfu/mL) carried out on potato dextrose broth (Himedia), and incubated on a shaker (ZHWY 211C, Zhicheng, Shanghai, China) under a vibration amplitude of 180 rpm for 96 h at + 28 °C.

### Yeast Cells and Dry Cell Mass Determination

The number of yeast cells in the culture was determined using a hemocytometer (Neubauer improved, Poly-Oprik, Bad Blankenburg, Germany). Yeast cells were harvested by centrifugation at 24000×*g* for 15 min at 4 °C (Heraeus, Biofuge Stratos, Osterode, Germany). The resulting cell pellet was washed twice with 0.9% NaCl and dried at 80 °C to obtain constant weight. The total cell mass was determined gravimetrically (g/L).

### Cellular Lipid Extraction, Purification, and Fractionation

Total lipids were extracted in a mixture of chloroform and methanol (Sigma-Aldrich, Darmstadt, Germany) (2:1, v/v) according to the method by Folch et al. [41], modified for lipid extraction by oleaginous yeasts as described by Dourou et al. [39]. The extracts were filtered through Whatman No. 1 filter paper and washed with a 0.88% KCl (Sigma-Aldrich, Darmstadt, Germany) solution to remove the nonlipid components. The washed extracts were then dried over anhydrous Na_2_SO_4_ (Sigma-Aldrich, Warsaw, Poland), and the solvents present in them were evaporated under vacuum using a Rotavapor R-20 device (BÜCHI AG, Flawil, Switzerland). Total cellular lipids were determined gravimetrically, and the percentage of lipids in the dry cell mass (L/*x*%) was calculated by dividing the total lipids (L, g/L) by the total cell mass (*x*, g/L), determined as described above.

The total lipids (about 100 mg) dissolved in 1 mL of chloroform were fractionated using a chromatographic column (25 mm × 100 mm) containing 1 g of silicic acid (Merck) activated at 80 °C for 12 h. By the successive application of dichloromethane (100 mL; Sigma-Aldrich, Darmstadt, Germany), acetone (100 mL; Merck), and methanol (50 mL), fractions containing neutral lipids (NL), glycolipids plus sphingolipids (G+S), and phospholipids (P), respectively, were obtained.

### Fatty Acid Analysis

FA moieties of total cellular lipids were transformed into their FA methyl esters (FAMEs) in a two-step reaction [32]. FAMEs were then analyzed using an Agilent 7890A gas chromatography system (Agilent Technologies, Shanghai, China) equipped with a flame ionization detector (FID) using an HP-88 column (60 m × 0.32 mm) (J&W Scientific, Agilent Technologies, Folsom, USA). Helium was used as a carrier gas at a flow rate of 1 mL/min. The injection temperature was maintained at 250 °C, the oven temperature at 200 °C, and the FID temperature at 280 °C. The obtained FAMEs were identified by comparison with standards.

The degree of FA unsaturation (unsaturation index (U.I.)) was calculated in ∆/mol from the equation:

$$ \mathrm{U}.\mathrm{I}.=\left[\%\mathrm{monoene}+2\times \left(\%\mathrm{diene}\right)\right]/100 $$

### Sugar Determination in the Medium

Glucose concentration in the growth medium was determined according to 3,5-dinitrosalicylic acid (DNS) method as described by Bzducha-Wróbel et al. [42] and expressed in g/L.

### Microscopy

The morphological structures of the yeast cells were analyzed using a Carl Zeiss optical microscope (GmbH, Gottingen, Germany), equipped with a digital camera (Exwave HAD, Sony, Tokyo, Japan) and an image analysis system (Pinnacle Studio 10).

The visualization of the lipid droplets (LDs) inside the yeast cells was performed as follows: 0.1 g of wet cell mass suspended in 1 mL of water was mixed with the Nile Red fluorescent reagent (C_20_H_18_N_2_O_2_) (Sigma-Aldrich, Darmstadt, Germany) suspended in absolute ethanol (0.1 mg/mL). The mixture was incubated for 1 h at room temperature in the dark. After incubation, the cells were washed twice with distilled water and centrifuged (4500×*g*, 10 min, 4 °C). The supernatant was discarded, and using the obtained cell pellet, samples for microscopic analysis were prepared. The LDs in yeast cells were visualized using an Axiostar 40 fluorescence microscope (Zeiss, Cambridge, United Kingdom), equipped with a 470/40 nm excitation filter, a ProgRes camera (Jenoptik CF Cool, Jena, Germany), and an image analysis system (ProgRes CapturePro_v2.7.7).

### Statistical Analysis

The data obtained from this study were subjected to analysis of variance using Statistica 13.3 program (StatSoft Inc., Tulsa, Oklahoma, USA). The significance of differences between the mean values in each group was tested by Tukey’s test at a significance level of *α* = 0.05.

## Results and Discussion

### Effect of Selenium on the Growth of *Y. lipolytica*

The unique features of *Y. lipolytica*, in combination with the availability of genetic tools for this species, have stimulated attention in the use of this yeast as a model microorganism, with great potentials for microbial-based biotechnology [36]. In this study, the physiological behavior of *Y. lipolytica* ACA DC 50109 and the so-referred here as “ALE_70” strain of *Y. lipolytica* [32] on the presence of Se at final concentrations (i.e., 10 mg/L) was tested. Growth of *Y. lipolytica* strains on 10 mg/L of Se was studied after 48, 72, and 96 h and compared with cultures performed on control media, without the presence of Se (i.e., 0 mg/L). This result is in agreement with previous studies, reporting that media containing Se in high concentrations inhibited the growth of other yeast strains [5, 16].

**Fig. 1:**
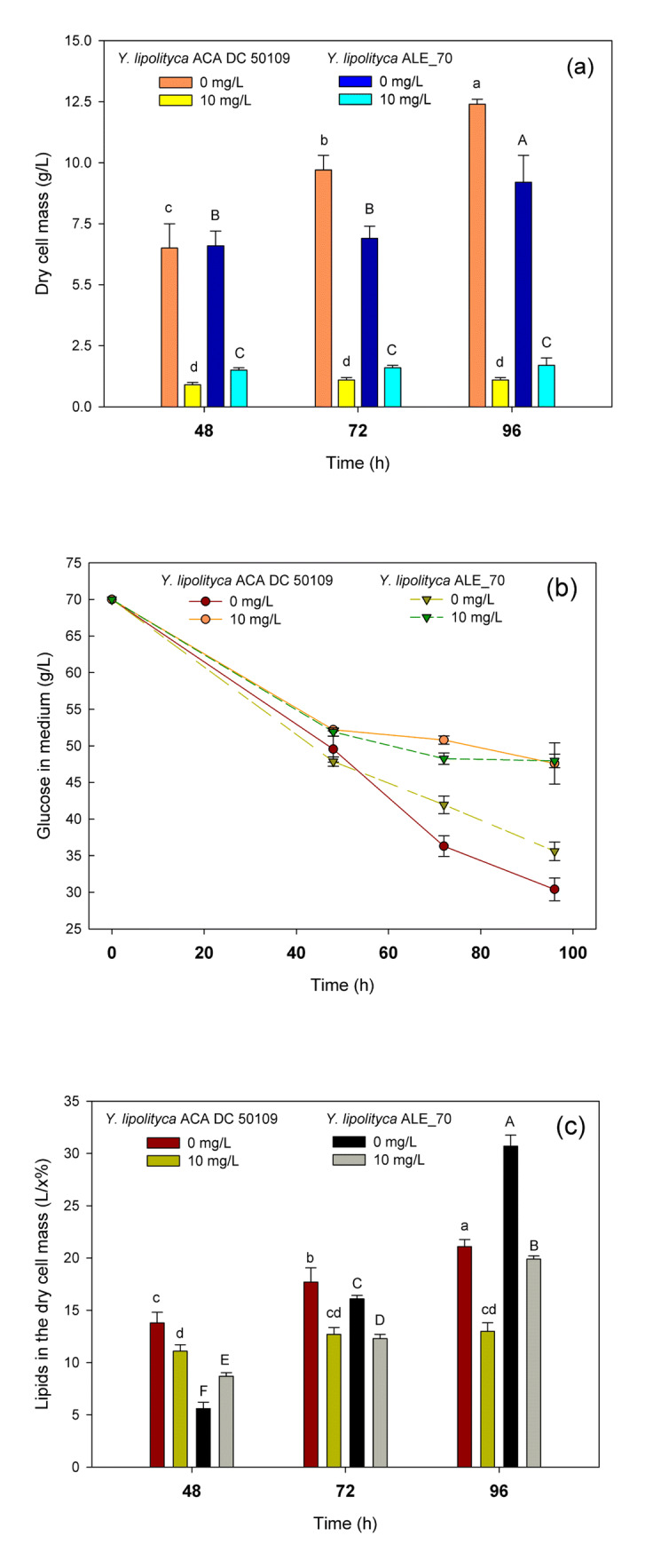
Growth (**a**), glucose consumption (**b**), and lipid accumulation (**c**) of *Y. lipolytica* strains cultivated in media containing different concentrations of Se (i.e., 0 and 10 mg/L)

The ALE_70 strain used in this present study has been obtained after adaptive laboratory evolution methodology, and its physiological behavior cultivated on glucose 70 g/L has been fully characterized and reported by Daskalaki et al. [32]. According to our results, the growth of *Y. lipolytica* ACA DC 50109 and ALE_70 obtained after 48 h of cultivation in the control medium were at a comparable level (statistically insignificant) since 6.5 and 6.6 g/L of dry cell mass were produced, respectively (Fig. 1a). These results are in agreement with Daskalaki et al. [32]. However, after 96 h of cultivation, the produced dry cell mass was higher of the ACA DC 50109 strain (i.e., 12.4 g/L) than the ALE_70 (i.e., 9.2 g/L), probably due to the lowest glucose assimilation rate that the second strain presented. When Se was present in the growth medium, the growth of both strains was negatively affected. Specifically, concerning the ACA DC 50109 strain, since the first 48 h, the biomass yield per unit of substrate (*Y*_X/glc_) was decreased from 0.32 to 0.05 g/g, resulting in 0.9 g/L of dry cell mass. In the case of ALE_70 strain, the presence of Se in the growth medium resulted at an 83% reduced cell mass compared with the cultivation in the control medium after 48 h and the *Y*_X/glc_ decreased from 0.30 to 0.08 g/g. The differences in the dry cell mass between the experimental and control media were statistically significant. In addition, although Daskalaki et al. [32] reported that the glucose assimilation rate remained unchanged for the evolved populations cultivated on glucose, it seems that Se strongly influenced glucose uptake for the ACA DC 50109 strain, compared with the ALE_70 (Fig. 1b). Probably, as the ALE_70 strain was obtained after ALE-based methodology, which relies on natural selection, performed on double-limited media, it could be attributed to differences in the metabolic properties of these two strains.

As it is mentioned above, inhibitory effects of Se are also reported for *Candida utilis* ATCC 9950 and *Saccharomyces cerevisiae* ATCC MYA-2200 strains [15]. The yeast *S. cerevisiae* has been used as a model microorganism by plenty of researches, in order to test toxicity and mutagenicity caused by Se and/or other metalloid elements [43]. According to literature, Se causes double-stranded DNA damage on *S. cerevisiae* [14, 44] and reduction of glutathione (GSH) [12, 45]. Some references indicated that *S. cerevisiae* exports extracellularly GSH during arsenite and tellurium stress, aiming a comprehensive binding and detoxification of these harmful metalloids [46, 47]. On the contrary, recently, Zhang et al. [48] reported that the presence of Se inhibits the cell growth of *C. utilis* CCTCC M 209298, but in parallel causes increment of GSH biosynthesis. The authors suggest that Se decreased carbon fluxes towards cell mass formation, but increased fluxes towards amino acids for the biosynthesis of GSH and related proteins. Most of the research on Se-enriched yeasts is based on its incorporation into proteins, replacing sulfur, and the effect on their structure and catalytic functions [4]. El-Bayoumy et al. [49] studying the influence of Se on the global protein expression of Se-enriched *S. cerevisiae* reported that Se resulted in the upregulation of a variety of proteins, including pyruvate kinase, elongation factor 2, and the heat shock protein 70, while TSI was downregulated. TSI is a key enzyme in glycolysis, and thereby essential for energy production. As the authors reported, a variety of metabolic processes are affected by Se, such as glycolysis, ATP binding, metal binding, nucleoside and nucleotide binding, protein structure, stress, and cell signaling pathways.

### Effect of Selenium on the Lipid Content and Lipid Fractions

Analyzing the diversity of the individual groups of microorganisms growing under stress conditions is of great importance in scientific research. In particular, due to their ability to accumulate high amount of TAGs utilizing various carbon sources, oleaginous yeasts are considered interesting subjects in biotechnological studies [50]. The ALE_70 strain used in this study was obtained after an alternative strategy, which relied on the evolution of *Y. lipolytica* ACA DC 50109 under alternating environments that encourages storage lipid synthesis. As a result, this strain is able to accumulate more lipids than the ACA DC 50109 after 77 generations [32]. According to results from the current investigation, after 96 h of cultivation in the control medium (i.e., 0 mg/L of Se), the ACA DC 50109 strain accumulated 22.0% lipids in the dry cell mass, while the ALE_70 strain accumulated 30.7% (Fig. 1c and Table 1) and consequently, our results are in agreement with Daskalaki et al. [32]. In addition, Dourou et al. [39] reported that maximum lipid accumulation of the ACA DC 50109 strain was 30% lipids in the dry cell mass, cultivated in a medium containing 40 g/L of glucose and various nutrients. The presence of Se in the growth medium negatively affected lipid accumulation in both yeast strains, while yet again as in the case of growth, the ACA DC 50109 was more affected. Specifically, the ACA DC 50109 strain was able to accumulate 41% less lipids in the dry cell mass (i.e., L/*x*% = 13.0%) after 96 h of cultivation, while the ALE_70 strain accumulated 21% less lipids, as the L/*x*% was 24.2% (Table 1).

**Table 1:** Lipid content in dry cell mass (L/*x*%), lipid fractions of neutral lipids (NL), glycolipids plus sphingolipids (G+S), and phospholipids (PL) in total lipids, and NL/polar ratio observed during 96-h culturing of *Y. lipolytica* strains in the presence of various concentrations of selenium

*Y. lipolytica* strain	Selenium (mg/L)	L/*x*(%)	Lipid fraction (%) in total lipids			NL/polar (w/w)
			NL	G+S	PL	
ACA DC 50109	0	21.1 ± 1.6^a*^	84.2 ± 2.07^a^	5.30 ± 0.17^d^	8.80 ± 0.92^d^	6.0
	10	13.0 ± 3.2^b^	40.8 ± 2.08^b^	26.5 ± 2.86^c^	30.6 ± 0.99^c^	1.4
ALE_70	0	30.7 ± 4.3^a^	62.5 ± 0.88^a^	12.5 ± 0.80^d^	13.2 ± 0.60^d^	2.4
	10	24.2 ± 2.7^b^	48.5 ± 1,77^b^	26.5 ± 2.85^c^	27.9 ± 1.42^c^	0.9

^*^Means with the same letters ^a,b,c^… considering one strain of yeasts are not significantly different (acc. Tukey’s HSD test)

ROS are generated as a natural by-product of metabolism in aerobic microorganisms. However, environmental stress (e.g., the presence of Se in the growth medium) can induce the production of ROS, such as hydrogen peroxide, in yeast cells. Usually, ROS cause lipid peroxidation, DNA damage, protein denaturation, and finally cell death. Lipid peroxidation and the reduction of ROS are performed in peroxisomes. The lipid peroxidation process and its products can change the structure and dynamics of membranes, and thus affect membrane fluidity and result in the destabilization of its structure [5]. Oxidative stress induces the oxidation of cellular thiol groups (–SH), causing rapid loss of the biological activity of proteins, while oxidation of thiol groups in the membrane can lead to its disintegration and increase its permeability. Though, microorganisms possess defense mechanisms (enzymatic and non-enzymatic), which protect them, to a certain extent, against the action of ROS [51]. Catalase in peroxisomes is the only antioxidative enzyme present in these organelles in yeasts. Recently, the role of mannitol in stress tolerance in microorganisms has been reported [52]. It is suggested, for the W29 strain of *Y. lipolytica*, that mannitol could be intracellularly accumulated in cells growing under stress conditions, involving in scavenging of generated ROS [53]. Lately, it has been suggested that ROS accumulation enhances lipid synthesis in oleaginous microorganisms [54]. However, the opposite trend was observed for *Y. lipolytica* strains and Se in the current investigation.

Fractionation of total lipids was performed on the last day of the breeding (i.e., 96 h), showing that, independently on the strain and the presence of Se, NL was the most abundant fraction, followed by PL and G+S (Table 1). This lipid distribution is similar to published results for *Y. lipolytica* [25, 32, 55]. However, for both *Y. lipolytica* strains, the presence of Se inhibited NL accumulation, and consequently the percentage of polar lipids (G+S, PL) on total lipids increased. Specifically, NL decreased by half in the lipids of the Se-enriched ACA DC 50109 strain and a 5-fold and an almost 4-fold increase in the percentage of G+S and PL, respectively, were observed (Table 1). High PL content is typical of cells growing in media that do not favor lipid accumulation and TAG synthesis. In addition, although the NL/polar ratio was 1.4 on the lipids of the Se-enriched ACA DC 50109 strain, interestingly on the lipids of Se-enriched ALE_70, the ratio was 0.9, showing that Se, apart from the inhibition of NL synthesis, provoked also the accumulation of polar lipids for this strain. The physiological role of NL is energy storage and provision, depending on the cell needs. The inhibition of NL accumulation observed in this study on the presence of Se could be explained by the fact that under stress conditions, cells tend to alter their membrane compositions (e.g., by synthesizing more polar lipids), as a protective mechanism for the cytosol against the environmental stress. Polar lipids are mainly membrane component of cells and organelles, assuring the transport and exchange of materials, and playing an important role in the modulation of the membranes [55]. According to Vázquez et al. [56], Se largely contributes to the integrity of the cell wall under adverse culture conditions.

### Effect of Selenium on the Fatty Acid Composition

FA composition of the total lipids of *Y. lipolytica* strains is presented in Table 2. Both strains grown in the control media showed the same FA profile: Oleic acid (C18:1 n-9) was the dominant FA, representing 52–55% of total FAs, followed by palmitic (C16:0) and palmitoleic (C16:1 n-7) acids, while stearic acid (C18:0) was also found in considerable concentrations. The FA profile of both strains was similar to that reported in previous investigations [25, 32, 55]. However, according to our results, the influence of Se on FA composition is strain-dependent. Slight modifications have been observed on the FA profile of ALE_70 strain, as the percentage of SFAs remained unchanged and the percentage of MUFAs was reduced. Interestingly, the percentage of the unsaturated linoleic acid (C18:2 n-6) was almost doubled comparing with the control culture, resulting in increase of the U.I. (i.e., from 0.81 to 0.91). On the other hand, the ACA DC 50109 strain seems to be more influenced by Se, as the percentage of SFAs increased by 2-fold, resulting in reduction of MUFAs, while the percentage of C18:2 n-6 was almost unaffected. The above resulted in 28% decrease of the U.I. in total lipids. On the contrary to our results, Čertík et al. [17] reported that the presence of Se increased FA unsaturation of total lipids in red yeasts. In addition, Pádrová et al. [57], reported that increased concentration of iron nanoparticles in the culture medium significantly decreased the amount of SFAs in the lipids of seven yeast strains, including *Y. lipolytica*. Kieliszek et al. [58] indicated the presence of unusual FAs (e.g., margaric acid (C17:0), margaroleic acid (C17:1), and tricosylic acid (C23:0)), in the lipids produced by *C. utilis* ATCC 9950, as a response to Se, probably due to oxidation of long-chain FAs.

**Table 2:** Fatty acid composition and fatty acid unsaturation index of total lipids of *Y. lipolytica* strains observed after 96-h culturing in the presence of various concentrations of selenium

*Y. lipolytica* strain	Selenium (mg/L)	Fatty acid composition (%, w/w)							ΣSFAs	ΣMUFAs	U.I.
		C14:0	C16:0	C16:1	C18:0	C18:1 n-9	C18:2 n-6	Others^§^			
ACA DC 50109	0	0.17 ± 0.00^j*^	16.9 ± 0.09^d^	11.4 ± 0.01^e^	7.64 ± 0.02^g^	55.2 ± 0.12^a^	4.20 ± 0.28^h^	4.30 ± 0.07^h^	24.8	66.7	0.75
	10	1.42 ± 0.01^i^	36.0 ± 0.60^c^	1.86 ± 0.01^i^	8.66 ± 0.02^f^	43.9 ± 0.03^b^	3.97 ± 0.03^h^	4.10 ± 0.02^h^	46.1	45.8	0.54
ALE_70	0	0.15 ± 0.02^i^	15.3 ± 0.06^d^	15.0 ± 0.15^d^	5.48 ± 0.06^f^	52.5 ± 0.04^a^	6.49 ± 0.01^e^	4.90 ± 0.02^g^	20.9	67.7	0.81
	10	0.38 ± 0.08^i^	16.2 ± 0.26^c^	16.7 ± 0.25^c^	4.26 ± 0.04^h^	40.8 ± 0.04^b^	16.5 ± 0.03^c^	5.10 ± 0.02^g^	20.9	57.5	0.91

*SFAs* saturated fatty acids; *MUFAs* monounsaturated fatty acids; *U.I.* unsaturation index

^*^Means with the same letters ^a,b,c^… considering one strain of yeasts are not significantly different (acc. Tukey’s HSD test)

^§^Others are C10:0, C12:0, C14:1

Studying the ratios presented in Table 3, we can evaluate the effect of Se on enzymes (i.e., desaturases and elongase) involved in FA synthesis. Specifically, we can assume that the activity of Δ^12^ desaturase in the ALE_70 strain was induced by Se, as the C18:2/C18:1 ratio increased almost 4 times. However, Δ^9^ desaturase, as well as the stearoyl-CoA desaturase-1 (SCD-1), seems to be unaffected, for the same strain. On the other hand, SCD-1 of the ACA DC 50109 strain was strongly affected by Se, as the C16:1/C16:0 ratio was remarkable reduced (i.e., from 0.68 to 0.05). Interestingly, the elongation in both *Y. lipolytica* strains was negatively affected (see reduction of C18:0/C16:0 ratio). FA elongase is an ER-localized enzyme, catalyzing the FA elongation, consuming 2 molecules of NADPH per C2-unit of elongation [23]. Probably, as Se influences a variety of metabolic processes, the available reducing power in the Se-enriched cells is insufficient to support FA elongation. Moreover, C18/C16 ratio reduced for both *Y. lipolytica* strains, on the contrary of results reported for red yeasts [17].

**Table 3:** Ratios of product-to-precursor in total lipids of *Y. lipolytica* strains cultivated in various concentrations of selenium

*Y. lipolytica* strain	Selenium (mg/L)	C18:2/C18:1	C18:1/C18:0	C16:1/C16:0	C18:0/C16:0	C18/C16
ACA DC 50109	0	0.08	7.23	0.68	0.45	2.4
	10	0.09	5.08	0.05	0.24	1.5
ALE_70	0	0.12	9.59	0.99	0.36	2.1
	10	0.40	9.57	1.03	0.26	1.8

### Effect of Selenium on the Morphological Features of the Yeast and the Yeast Cell Mass

**Fig. 2:**
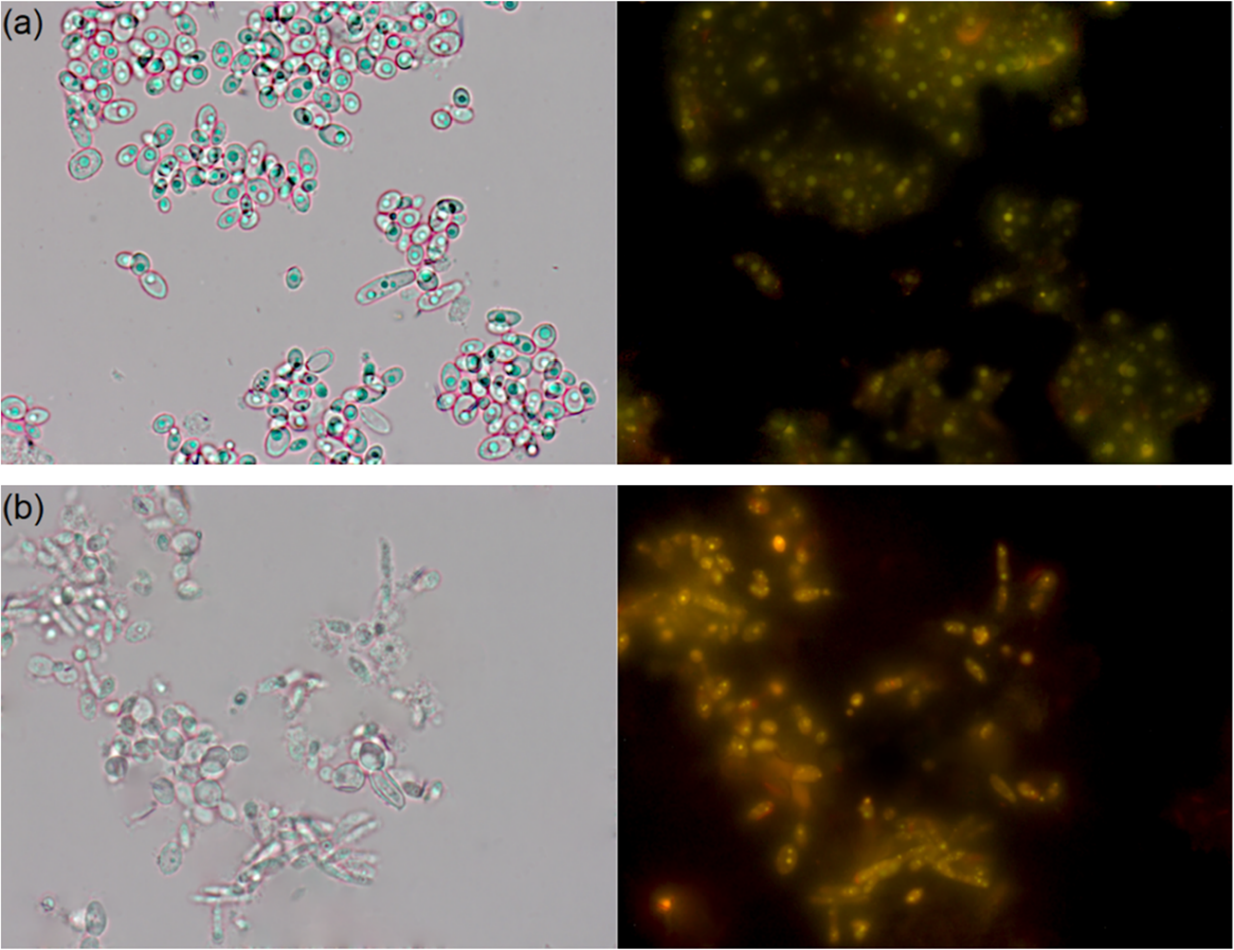
Morphology of the yeast cells and lipid droplets within the cells of *Yarrowia lipolytica* ACA DC 50109 strain under optical microscope (left) and fluorescence microscope (right) after staining with Nile Red, cultivating in medium without Se (**a**) and with Se (**b**)

**Fig. 3:**
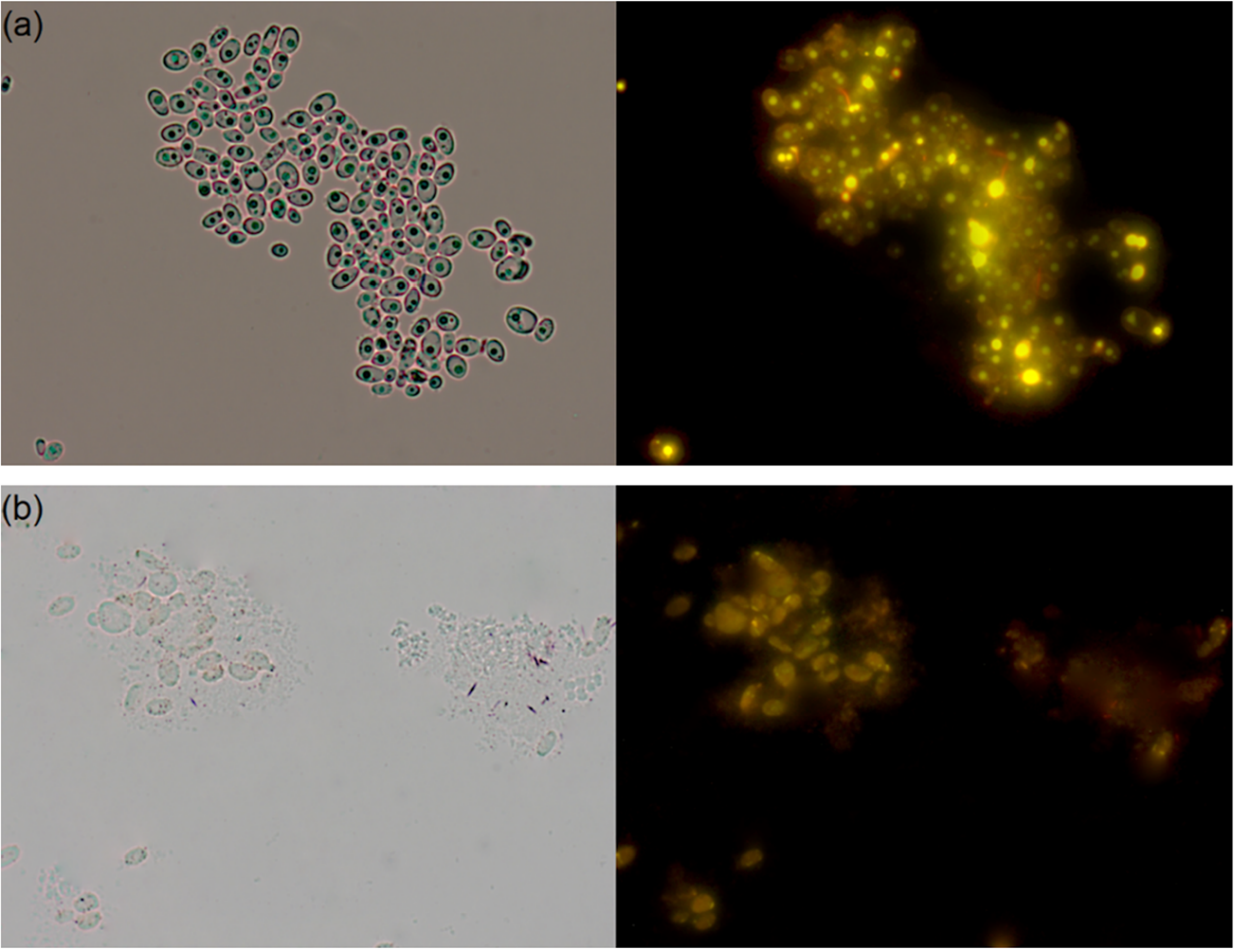
Morphology of the yeast cells and lipid droplets within the cells of *Yarrowia lipolytica* ALE_70 strain under optical microscope (left) and fluorescence microscope (right) after staining with Nile Red, cultivating in medium without Se (**a**) and with Se (**b**)

*Yarrowia lipolytica* is a dimorphic yeast, appearing in the form of yeast cells, mycelium, or pseudo-mycelium depending on the dissolved oxygen concentration [31]. During the growth on control medium, similar morphological features were observed. Both strains of *Y. lipolytica* were able to form yeast cells, which were round or slightly ellipsoidal, while LDs were clearly visible and they had different shapes depending on the size of the cells. Large LDs can occupy most of the cell, as it is depicted in the microscopic images (right pictures in Figs. 2a and 3a). Daskalaki et al. [32] reported that LDs from stem cells move to daughter cells, during *Y. lipolytica* reproduction through budding process. The addition of Se to the growth medium resulted in significant morphological changes in the yeast cells (Fig. 2b and 3b) compared with the control cultures. Most of the cells of the ACA DC 50109 strain appeared large and round, while the appearance of pseudo-mycelia was also notable. The cells of the ALE_70 strain had changes in the appearance of cell wall (membrane syndrome). As a result of NL biosynthesis inhibition, microscopic images showed the presence of smaller LDs in the cytosol of both yeast strains cultured in the presence of Se, compared with the control cultures. In addition, a common feature of the two Se-enriched strains was the presence of characteristic granules (yellowish LDs) in the entire cytosol region, as it was visualized under the fluorescence microscope (right pictures in Fig. 2b and 3b). However, further analysis needs to be performed to confirm these data.

It can be assumed that the addition of Se may have influenced the composition and density of cell membranes, leading to the appearance of pores. As a consequence, cell organelles are leaked to the extracellular environment. Zhang et al. [48] reported that sodium selenite present on a culture of *C. utilis* CCTCC M 209298 resulted on an increase of the permeability of yeast cells, as the expression of genes associated with the synthesis of the components present in the cytoplasmic membrane and cell wall were downregulated. The occurrence of cell wall folding and rearrangement of the yeast cell cytoskeleton may disturb the breakdown of tonoplasts and cause leakage of lytic enzyme and rapid cell contraction, thereby affecting the final size of yeast cells [6]. Cellular cytosol leakage could occur due to autolysis or cell autophagy. In addition, low-molecular-weight polyphosphate granules could be observed in the vacuoles, while the integrity of their membrane may be affected [59].

To date, no studies have been conducted on the uptake and transformation of Se by oleaginous yeasts. In the present study, we observed that the Se-enriched media showed a change in color from cream to light red, after cultivation of both *Y. lipolytica* strains. This color change of the cell suspension can be attributed to the metabolic activity of the yeast. A similar phenomenon has been described by Kieliszek et al. [16] and Tarze et al. [60], who indicated that the reduction of Se ions to elemental form leads to the development of red color. This can be considered one of the stages of detoxification of Se by the microorganisms. A study by Hamza et al. [61] showed that *Y. lipolytica* NCIM 3589 strain could tolerate up to 8.0 mM concentration of selenite sodium. The authors also obtained a red-colored yeast cell mass, which suggested the synthesis of Se in the form of nanoparticles (SeNP). However, the dose of Se used in the study negatively affected the growth compared with the control medium, which was also confirmed by the color of the obtained cell mass.

## Conclusion

The last few years, the study of Se metabolism by microorganisms and its effect on oxidative stress is under the microscope, as the demand for Se-enriched cell mass serving as a dietary supplement is increasing. The current scientific literature lacks information on the effect of Se on growth and lipid metabolism in oleaginous microorganisms. The current study is the first to our knowledge, which examined the effect of Se on the growth, lipid accumulation, and FA composition of the oleaginous yeast *Y. lipolytica*. Cell mass production and lipid accumulation were inhibited in the presence of Se, while lipid fractions and FA composition were differently affected among the two tested strains, reflecting the adaptation of the yeast in stress conditions. In addition, morphological changes in the cells were observed. It is of high interest to understand the mechanisms that microorganisms create so as to survive in adverse environmental conditions, and the current investigation is helpful in assessing not only the potential use of yeast cell mass enriched with deficit elements (such as Se) but also the lipid production processes that can be applied in various industries. We hope that our study encourages further research in order to expand our knowledge about the effect of this element on the lipid metabolism of various microorganisms.
